# Supplementary material for: External evaluation of the Dynamic Criticality Index: A machine learning model to predict future need for ICU care in hospitalized pediatric patients
Source: PLoS One. 2024 Jan 29;19(1):e0288233. doi: 10.1371/journal.pone.0288233 (PMC10824440; doi:10.1371/journal.pone.0288233)
Supplement: S1 File — All laboratory, vital sign, and demographic variables are outlined in S1 File. A summary of medication data is provided, further elaborated in S2 File. (PDF) [file pone.0288233.s001.pdf]

Supplemental Information 1: Independent Variables for Institutional Criticality Index-Dynamic Models

Table A. Independent variables.[1]

**Lab Variables**[1,3,4]				**Vital Signs**[1,3,4]	**Medications**[2,3]	**Other**
Albumin	Bilirubin Indirect	Hemoglobin	Platelets	BP-systolic	1113 individual medications	Age<br>Sex
ALT	Bilirubin Total	Hematocrit	Potassium	BP- diastolic	143 medication categories (6)	
Arterial Lactate	BUN	INR	Protime	Heart Rate		
PO2 (arterial)	Calcium	Glucose	Sodium	Respiratory Rate		
AST	Calcium Ionized	PTT	Total Protein	Temperature		
Base Excess	Chloride	PCO2[5]	Venous Lactate	Coma Score		
Bicarbonate	Creatinine	pH[5]	WBC			
Bilirubin Direct	Fibrinoge n					

1. Summarized for modeling with the following statistics for each variable: the count, sample mean, sample standard deviation (0 if the count was <2), maximum, and minimum. There were a total of 934 variables used for modeling: 300 derived from the 30 laboratory variables, 60 from the 6 vital signs, and 572 from the 143 medication categories, sex and age of patient at admission.
2. Summarized for modeling with the following statistics: the 6-hour sum per medication category of the number of medications given each hour; 2) the count of the previous time periods per medication category that the patient received one or more medications; 3) the proportion of the previous time periods per medication category that the patient received one or more medications.
3. Therapeutic intensity is reflected in the number of vital sign and laboratory measurements and medications.
4. If during the first six-hour time period there were missing values, these values were adjusted to the median of the first six-hour time periods adjusted to the following age groups: <1week, 1week-<4weeks, 4weeks-<3months, 3months-<1year, 1year-<2years, 2years-<3years, 3years-<8years, 8years-<12years, 12years-<22years.
5. Arterial, venous, capillary.
6. Classified by Multum[1]
