# Supplementary material for: External evaluation of the Dynamic Criticality Index: A machine learning model to predict future need for ICU care in hospitalized pediatric patients
Source: PLoS One. 2024 Jan 29;19(1):e0288233. doi: 10.1371/journal.pone.0288233 (PMC10824440; doi:10.1371/journal.pone.0288233)
Supplement: S2 File — Details of medication classification are included in S2 File. (PDF) [file pone.0288233.s002.pdf]

Supplemental Information 2: Medication Data

The Children's National Database contains the drug name (generic and brand), national drug code (NDC), administration times, and administration frequency. Medications were assumed to be administered to patients during the times the pharmacy dispensed them to be administered. The duration of medication administration was rounded to the nearest hour. If a single dose of medication was administered in an hour – the patient was considered to have received one hour of medication.

To assess medications by class, the medication table was linked to an industry standard for medication classification, Multum™ , via the NDC number.[1,2] Multum™ provides information about medications therapeutic action in three categories from general mechanism of action to a specific therapeutic category. Table B features the 144 medication categories that were utilized Multum™ to categorized 96% of medications recorded in Health Facts®. For medications present in multiple Multum™ categories, a prioritization list was developed to classify each medication in the category most relevant to ICU care (Table C).

Some medication categories were combined if they had similar mechanisms of action or therapeutic usage. Inotropic agents, vasopressin, vasopressors and vasopressin antagonists were combined as their mechanism of action targets blood pressure elevation and they often have varying effects at different doses. Immunosuppressive agents, anti-neoplastic and anti-rheumatics were combined as all medications target reducing the body's innate immune system. All anti-cholinergic categories (anti-Parkinson agents, chronotropic agents and anti-spasmodic agents) were combined due to similar mechanisms of action and the inability to determine which therapeutic purpose they were employed for. Inhaled nitric oxide (iNO) is currently not FDA approved for pulmonary hypertension in pediatric patients; however, it is used for this purpose or similar physiologic reasons in the ICU. Therefore, iNO was included in the "agents for pulmonary hypertension" category. Muscle relaxants and neuromuscular blockade agents were combined as several drugs are present in both categories. Erythropoiesis stimulating agents and colony stimulating agents were combined as both promote stimulation of cell lines. Cardio-selective and non-cardio selective) beta blockers were combined due to their similar mechanism of action and therapeutic purpose. Anti-depressants and antipsychotics were also combined as both are psychotherapeutic agents employed in the ICU.

The drugs not categorized by Multum™ were categorized via independent assessment by three physicians (AKP, MMP) into the existing categories by their mechanism of action and/or therapeutic purpose (Table D).

Table B. Original Multum™ Categories Applied to Institutional Medication Data

**Multum™ Categories**
Ace inhibitors
Ace inhibitors with thiazides
Adrenal cortical steroids
Agents for hypertensive emergencies
Agents for pulmonary hypertension
Analgesics
Angiotensin ii inhibitors
Anorectal preparations
Anti infective
Antiadrenergic agents centrally acting
Antiadrenergic agents peripherally acting
Antianginal agents
Antiarrhythmic agents
Anticholinergic antiparkinson agents
Anticholinergic chronotropic agents
Anticholinergics antispasmodics
Anticoagulant reversal agents
Anticoagulants
Anticonvulsants
Antidepressants
Antidiabetic agents
Antidiuretic hormones

Antidotes
Antiemetic antivertigo agents
Antigout agents
Antihistamines
Antihyperlipidemic agents
Antihypertensive combinations
Antihyperuricemic agents
Antineoplastics
Antiplatelet Agents
Antipsychotics
Antirheumatics
Antiseptic And Germicides
Antitussives
Anxiolytics sedatives and hypnotics
Beta blockers cardioselective
Beta blockers non-cardioselective
Biologicals
Bisphosphonates
Bronchodilators
Calcium Channel Blocking Agents
Cation Exchange Resins
Cerumenolytics
Chelating Agents
Cholinergic Agonists

Cholinergic Muscle Stimulants
Cholinesterase Inhibitors
CNS Stimulants
Coagulation Modifiers
Decongestants
Diuretics
Dopaminergic antiparkinsonism agents
Erythropoiesis stimulating agents
Expectorants
Gastrointestinal agents
General anesthetics
Glucose elevating agents
Hormones hormone modifiers
Immunologic agents
Immunosuppressive agents
Inotropic agents
Intravenous nutritional products
Ionic iodinated contrast media
Iron products
Leukotriene modifiers
Local injectable anesthetics
Lung surfactants
Magnetic resonance imaging contrast media
Minerals and electrolytes

Miscellaneous bone resorption inhibitors
Miscellaneous cardiovascular agents
Miscellaneous central nervous system agents
Miscellaneous coagulation modifiers
Miscellaneous diagnostic dyes
Miscellaneous genitourinary tract agents
Miscellaneous ophthalmic agents
Miscellaneous respiratory agents
Miscellaneous topical agents
Miscellaneous uncategorized agents
Miscellaneous vaginal agents
Mouth and throat products
Muscle relaxants
Mydriatics
Narcotic analgesics
Nasal antihistamines and decongestants
Nasal lubricants and irrigations
Nasal steroids
Neuromuscular blocking agents
Nitric oxide
Non iodinated contrast media
Non ionic iodinated contrast media
Nutraceutical products
Ophthalmic anesthetics

Ophthalmic anti -infectives
Ophthalmic anti-inflammatory agents
Ophthalmic antihistamines and decongestants
Ophthalmic diagnostic agents
Ophthalmic glaucoma agents
Ophthalmic lubricants and irrigations
Ophthalmic steroids
Ophthalmic steroids with anti-infectives
Ophthalmic surgical agents
Oral nutritional supplements
Other
Otic anti-infectives
Otic steroids with anti-infectives
Peripheral vasodilators
Phosphate binders
Plasma expanders
Platelet stimulating agents
Probiotics
Respiratory inhalant products
Sclerosing agents
Smoking cessation agents
Sterile irrigating solutions
Topical acne agents
Topical anesthetics

Topical anti-infectives
Topical anti -rosacea agents
Topical antibiotics
Topical antifungals
Topical antihistamines
Topical antipsoriatics
Topical antivirals
Topical astringents
Topical debriding agents
Topical emollients
Topical non-steroidal anti-inflammatories
Topical rubefacient
Topical steroids
Topical steroids with anti-infectives
Ultrasound contrast media
Upper respiratory combinations
Urea cycle disorder agents
Urinary antispasmodics
Urinary pH modifiers
Uterotonic agents
Vaginal anti-infectives
Vasodilators
Vasopressin antagonists
Vasopressors

Vitamin and mineral combinations
Vitamins

Table C: Medication Prioritization

Neuromuscular Blockade Agents
Vasopressor
Inotropic agents
Anxiolytics, Sedatives and Hypnotics
Analgesics
Antiarrhythmic agents
General Anesthetics
Anticonvulsant
Agents for hypertensive emergencies
Beta blockers, cardio-selective
Beta blockers, non-cardio-selective
Beta blockers with thiazides
Calcium Channel Blocking agents
Ace-inhibitor with thiazides
Ace-inhibitor with calcium channel blocking agents
Angiotensin converting enzyme (ACE) inhibitor
Angiotensin II inhibitor
Angiotensin II inhibitor with thiazide,
Angiotensin receptor blockers and neprilysin inhibitors
Angiotensin II inhibitors with calcium channel blockers

Agents for pulmonary hypertension
Anticholinergic chronotropic agents
Lung surfactants
Anticoagulant
Minerals and electrolytes
bronchodilators
Analgesics
Antiplatelet agents
Diuretics
Antidiabetic agents
Anti-infectives
Adrenal cortical steroids
Hormones/hormone modifiers
Diuretics
Anti-arrhythmic agents
antihypertensive combinations
Anti-neoplastic
Antidepressant
gastrointestinal agents
anticholinergics/antispasmodics
Antihistamine
Immunosuppressive agents
Immunologic agents
Colony stimulating factors

Antihyperuricemic agents
Antirheumatics
Respiratory inhalant products
Antihistamine
Hormone/hormone modifiers
Antiemetic/antivertigo agents
Urinary antispasmodic
Intravenous nutritional products
Vasodilators
Plasma expanders
Anticoagulants
Antiadrenergic agents, centrally acting
Cholinergic muscle stimulants
CNS stimulants
Antidotes
Erythropoiesis-stimulating agents
Miscellaneous cardiovascular agents
Antidiuretic hormones
Glucose elevating agents
Muscle relaxants
Antiarrhythmic agents
Vasopressin antagonists
Antihyperlipidemic agents
Biologicals

Antianginal agents
Antineoplastics
Cholinesterase inhibitors
Nitric oxide
Vasopressin

Table D: Manually Categorized Medications

Drug Name	First Tier Category Name	Second Tier Category Name	Third Tier Category Name
5-aminosalicylic acid	5-aminosalicylates	gastrointestinal agents	
Amrinone	inotropic agents	cardiovascular agents	
Botulism antitoxin bivalent (Equine) A and B	antitoxins and antivenins	biologicals	
Bretylium	group III antiarrhythmics	antiarrhythmic agents	cardiovascular agents
Carbenicillin	antipseudomonal penicillins	penicillins	anti-infectives
Carbetocin	uterotonic agents	genitourinary tract agents	
Cefamandole	second generation cephalosporins	cephalosporins	anti-infectives
Cefonicid	second generation cephalosporins	cephalosporins	anti-infectives
Ceftizoxime	third generation cephalosporins	cephalosporins	anti-infectives
Cephalothin	first generation cephalosporins	cephalosporins	anti-infectives
Cilazapril	angiotensin converting enzyme (ACE) inhibitors	cardiovascular agents	

Cilazapril-hydrochlorothiazide	ACE inhibitors with thiazides	antihypertensive combinations	cardiovascular agents
Cloxacillin	penicillinase resistant penicillins	penicillins	anti-infectives
Danaparoid	heparins	anticoagulants	coagulation modifiers
Digitoxin	inotropic agents	cardiovascular agents	NULL
Dirithromycin	macrolides	macrolide derivatives	anti-infectives
Doxacurium	neuromuscular blocking agents	muscle relaxants	central nervous system agents
Elvitegravir	integrase strand transfer inhibitor	antiviral agents	anti-infectives
Enalapril-felodipine	ACE inhibitors with calcium channel blocking agents	antihypertensive combinations	cardiovascular agents
Enflurane	general anesthetics	central nervous system agents	NULL
Fenoterol	adrenergic bronchodilators	bronchodilators	respiratory agents
Fenoterol-ipratropium	bronchodilator combinations	bronchodilators	respiratory agents
Fomivirsen	miscellaneous antivirals	antiviral agents	anti-infectives
Fospropofol	general anesthetics	central nervous system agents	NULL
Halofantrine	miscellaneous antimalarials	antimalarial agents	anti-infectives
Halothane	general anesthetics	central nervous system agents	NULL
Insulin	insulin	antidiabetic agents	metabolic agents
Insulin isophane	insulin	antidiabetic agents	metabolic agents

Insulin zinc	insulin	antidiabetic agents	metabolic agents
Insulin zinc extended	insulin	antidiabetic agents	metabolic agents
Interferon alfa-2a	antineoplastic interferons	antineoplastics	NULL
Interferon alfa-2b-ribavirin	antiviral combinations	antiviral agents	anti-infectives
Interferon alfacon-1	interferons	immunostimulants	immunologic agents
Interferon alfa-n1	interferons	immunostimulants	immunologic agents
Iodoquinol	amebicides	anti-infectives	NULL
Ipratropium-salbutamol	bronchodilator combinations	bronchodilators	respiratory agents
Isoetharine	adrenergic bronchodilators	bronchodilators	respiratory agents
Kanamycin	aminoglycosides	anti-infectives	NULL
Ketotifen	ophthalmic antihistamines and decongestants	ophthalmic preparations	topical agents
Lipid surfactant	lung surfactants	respiratory agents	
Lomefloxacin	quinolones	anti-infectives	NULL
Loracarbef	second generation cephalosporins	cephalosporins	anti-infectives
Lucinactant	lung surfactants	respiratory agents	NULL
Lymphocyte immune globulin, anti-thymocyte	selective immunosuppressants	immunosuppressive agents	immunologic agents
Magaldrate	antacids	gastrointestinal agents	NULL

Mephobarbital	barbiturates	anxiolytics, sedatives, and hypnotics	central nervous system agents
Mephobarbital	barbiturate anticonvulsants	anticonvulsants	central nervous system agents
Metaraminol	vasopressors	cardiovascular agents	NULL
Methoxyflurane	general anesthetics	central nervous system agents	NULL
Moricizine	group I antiarrhythmics	antiarrhythmic agents	cardiovascular agents
Muromonab-CD3	selective immunosuppressants	immunosuppressive agents	immunologic agents
Nadroparin	heparins	anticoagulants	coagulation modifiers
Nedocromil	mast cell stabilizers	respiratory inhalant products	respiratory agents
Netilmicin	aminoglycosides	anti-infectives	NULL
Nicoumalone	coumarins and indanediones	anticoagulants	coagulation modifiers
Nitrazepam	benzodiazepine anticonvulsants	anticonvulsants	central nervous system agents
Nitroprusside	agents for hypertensive emergencies	cardiovascular agents	
Norfloxacin	quinolones	anti-infectives	NULL
Nylidrin	peripheral vasodilators	cardiovascular agents	
Oprelvekin	interleukins	immunostimulants	immunologic agents
Oprelvekin	platelet-stimulating agents	coagulation modifiers	NULL

Orciprenaline	adrenergic bronchodilators	bronchodilators	respiratory agents
Oxprenolol	beta blockers, non-cardioselective	beta-adrenergic blocking agents	cardiovascular agents
Paraldehyde	miscellaneous anxiolytics, sedatives and hypnotics	anxiolytics, sedatives, and hypnotics	central nervous system agents
Peginesatide	erythropoiesis-stimulating agents	biologicals	NULL
Peginterferon alfa-2b-ribavirin	antiviral combinations	antiviral agents	anti-infectives
Penbutolol	beta blockers, non-cardioselective	beta-adrenergic blocking agents	cardiovascular agents
Penicillin	natural penicillins	penicillins	anti-infectives
Pentastarch	miscellaneous uncategorized agents	miscellaneous agents	NULL
Pipecuronium	neuromuscular blocking agents	muscle relaxants	central nervous system agents
Piperacillin	antipseudomonal penicillins	penicillins	anti-infectives
Pirbuterol	adrenergic bronchodilators	bronchodilators	respiratory agents
Pivampicillin	aminopenicillins	penicillins	anti-infectives
Pivmecillinam	natural penicillins	penicillins	anti-infectives
Pizotifen	migraine treatment		
Plicamycin	antineoplastic antibiotics	antineoplastics	NULL
Poractant	lung surfactants	respiratory agents	
Proguanil	miscellaneous antimalarials	antimalarial agents	anti-infectives
Ralitrexed	folate antimetabolite for chemotherapy		
Rapacuronium	neuromuscular blocking agents	muscle relaxants	central nervous system agents

Respiratory syncytial virus immune globulin	immune globulins	immunologic agents	NULL
Reteplase	thrombolytics	coagulation modifiers	NULL
Salbutamol	adrenergic bronchodilators	bronchodilators	respiratory agents
Simvastatin-sitaGLIPtin	antihyperlipidemic combinations	antihyperlipidemic agents	metabolic agents
Sparfloxacin	quinolones	anti-infectives	NULL
Spectinomycin	miscellaneous antibiotics	anti-infectives	NULL
Spiramycin	macrolides	macrolide derivatives	anti-infectives
Stanozolol	androgens and anabolic steroids	sex hormones	hormones/hormo ne modifiers
Streptokinase	thrombolytics	coagulation modifiers	NULL
Sulfadiazine-trimethoprim	sulfonamides	anti-infectives	
Sulfamethizole	sulfonamides	anti-infectives	NULL
Sulfamethoxazole	sulfonamides	anti-infectives	NULL
Sulfapyridine	sulfonamides	anti-infectives	
Sulfisoxazole	sulfonamides	anti-infectives	NULL
Thioproperazine	phenothiazine antipsychotics	antipsychotics	psychotherapeutic agents
Thyrotropin alpha	thyroid hormones	hormones/hormo ne modifiers	
Ticarcillin	antipseudomonal penicillins	penicillins	anti-infectives
Ticarcillin-clavulanate	penicillins/beta-lactamase inhibitors	penicillins	anti-infectives
Trimetrexate	miscellaneous antibiotics	anti-infectives	NULL

1. UMLS Metathesaurus - MMSL (Multum) - Synopsis. Accessed July 25, 2018. https://www.nlm.nih.gov/research/umls/sourcereleasedocs/current/MMSL/

2. Fung KW, Kapusnik-Uner J, Cunningham J, Higby-Baker S, Bodenreider O. Comparison of three commercial knowledge bases for detection of drug-drug interactions in clinical decision support. *J Am Med Inform Assoc*. 2017;24(4):806-812. doi:10.1093/jamia/ocx010
