# Supplementary material for: External evaluation of the Dynamic Criticality Index: A machine learning model to predict future need for ICU care in hospitalized pediatric patients
Source: PLoS One. 2024 Jan 29;19(1):e0288233. doi: 10.1371/journal.pone.0288233 (PMC10824440; doi:10.1371/journal.pone.0288233)
Supplement: S3 File — All laboratory and vital sign data values imputed in development and validation of the Criticality Index are included in S3 File. (PDF) [file pone.0288233.s003.pdf]

Supplemental Information 3. Details of Imputation for Institutional Criticality Index-Dynamic Models

The initial time period required values for all laboratory and vital sign data. We imputed the values from the medians by age groups of those patients who had these measurements in the first time period. Note, that the imputed data also included the measurement count for the laboratory test or vital sign measurement which was set to 0 to indicate an imputed value. The age groups were a composite of the age groups used for display of normal data by various sources.

Table A. Percentage of initial time periods with imputed values.

**Laboratory Variables**	**Percentage**
Albumin	76.19
ALT	77.32
AST	77.28
PO2	98.64
Base Excess	88.48
Bicarbonate	59.73
Bilirubin Indirect	99.80
Bilirubin Total	76.39
BUN	59.55
Calcium	60.42
Calcium Ionized	89.24
Chloride	59.12
Creatinine	59.55
Fibrinogen	99.32
Glucose	56.42
Hematocrit	51.26
Hemoglobin	50.95
INR	94.45
Lactate Arterial	98.75
Lactate Venous	89.91
PCO2	88.48
pH	88.53
Platelets	54.61
Potassium	57.83
Protime	99.96
PTT	94.49
Sodium	56.98
Total Protein	76.79

White blood count	53.88
**Vital Signs**	
Blood Pressure Systolic	12.48
Blood Pressure Diastolic	12.59
Coma Score	82.65
Hearth Rate	0.29
Respiratory Rate	0.37
Temperature	6.18

ALT = alanine transaminase; AST = aspartate aminotransferase; BUN = blood urea nitrogen; INR = international normalized ratio.

Table B. Laboratory Data Imputed in the First Time Period. Values presented are medians.

**Age Groups**	**Albumin**	**ALT**	**Arterial pO2**	**AST**	**Base Excess**
1hr - <1wk	2.8	15	68	54.0	-4.0
1wk - <4wks	3.1	22	61.5	39.0	-0.2
4wks - <3mo	3.3	25	61	39.0	0.5
3mo - <1yr	3.6	26	77.5	43.0	-0.9
1yr - <2yrs	3.8	23	91	43.0	-3.9
2yrs - <3yrs	3.8	22	119	41.0	-2.0
3yrs - <8yrs	3.8	19	113	37.0	-2.0
8yrs - <12yrs	3.9	19	129	30.0	-2.8
12yrs - <22yrs	3.8	20	139	25.0	-3.0
	**Direct bilirubin**	**Indirect bilirubin**	**Total bilirubin**	**BUN**	**Calcium**
1hr - <1wk	0.4	6.0	7.3	9.0	9.0
1wk - <4wks	0.4	4.6	2.3	9.0	9.9
4wks - <3mo	0.3	0.9	0.5	9.0	9.8

3mo - <1yr	0.1	0.4	0.3	9.0	9.8
1yr - <2yrs	0.1	0.3	0.3	11.0	9.6
2yrs - <3yrs	0.1	0.4	0.3	11.0	9.5
3yrs - <8yrs	0.1	0.4	0.4	11.0	9.4
8yrs - <12yrs	0.2	0.5	0.4	11.0	9.3
12yrs - <22yrs	0.2	0.5	0.4	11.0	9.0
	**Ionized Calcium**	**Chloride**	**Creatinine**	**Fibrinogen**	**Glucose**
1hr - <1wk	1.2	106.0	0.6	201.5	72.0
1wk - <4wks	1.3	104.0	0.4	272.9	87.5
4wks - <3mo	1.3	105.0	0.3	265.0	94.5
3mo - <1yr	1.3	104.0	0.3	307.5	101.0
1yr - <2yrs	1.2	104.0	0.3	275.0	97.0
2yrs - <3yrs	1.2	104.0	0.3	262.0	96.0
3yrs - <8yrs	1.2	104.0	0.4	324.5	100.0
8yrs - <12yrs	1.2	103.0	0.5	333.5	104.0
12yrs - <22yrs	1.1	104.0	0.7	341.5	105.0
	**HCO3**	**Hematocrit**	**Hemoglobin**	**INR**	**Arterial Lactate**
1hr - <1wk	23.0	47.5	16.2	1.3	1.4
1wk - <4wks	24.0	39.6	13.6	1.3	1.2
4wks - <3mo	24.8	31.1	10.6	1.1	1.8
3mo - <1yr	22.5	33.8	11.4	1.3	1.0
1yr - <2yrs	21.0	34.3	11.5	1.2	1.2
2yrs - <3yrs	22.0	34.1	11.6	1.1	1.0

3yrs - <8yrs	23.0	34.5	11.8	1.2	1.7
8yrs - <12yrs	24.0	36.3	12.4	1.1	1.9
12yrs - <22yrs	24.0	37.3	12.6	1.2	2.2
	**Venous Lactate**	**Partial Thromboplastin Time (PTT)**	**pCO2**	**pH**	**Platelet Count**
1hr - <1wk	2.3	42.4	43.1	7.3	227.0
1wk - <4wks	3.2	39.0	44.3	7.4	338.0
[4wks,3mnths]	3.8	35.2	50.0	7.3	378.0
3mo - <1yr	2.2	32.0	45.0	7.4	339.0
1yr - <2yrs	1.8	29.0	39.6	7.4	313.0
2yrs - <3yrs	1.3	29.0	39.1	7.3	290.0
3yrs - <8yrs	2.0	29.0	39.0	7.4	274.0
8yrs - <12yrs	2.3	29.0	37.0	7.3	266.0
12yrs - <22yrs	2.2	28.4	36.0	7.3	239.0
	**Potassium**	**Pro Time**	**Sodium**	**Total Protein**	**White Blood Cell Count**
1hr - <1wk	4.6	15.1	139.0	5.2	13.9
1wk - <4wks	5.0	14.4	138.0	5.5	11.3
4wks - <3mo	5.0	13.8	138.0	5.5	10.9
3mo - <1yr	4.6	14.0	138.0	6.1	11.3
1yr - <2yrs	4.3	12.4	138.0	6.8	12.0
2yrs - <3yrs	4.2	12.4	138.0	6.8	10.4
3yrs - <8yrs	4.0	12.4	138.0	6.9	10.2

8yrs - <12yrs	4.0	12.4	138.0	7.1	9.7
12yrs - <22yrs	3.9	13.0	139.0	7.0	10.5

Table C. Vital Sign Values Imputed in the First Time Period. Values presented are medians.

Age Group	Systolic BP	Diastolic BP	Coma Score	Heart Rate	Respiratory Rate	Temperature (Centigrade)
1hr - <1wk	63	35	14	150	48	36.8
1wk - <4wks	76	45	14	159	41	36.9
4wks - <3mo	87	49	14	158	40	36.9
3mo - <1yr	99	57	14	148	38	36.8
1yr - <2yrs	107	64	15	141	30	37.1
2yrs - <3yrs	108	64	15	133	27	36.9
3yrs - <8yrs	106	64	15	120	24	37.0
8yrs - <12yrs	112	67	15	106	21	36.9
12yrs - <22yrs	120	70	15	96	19	36.8

Sources for Age References

https://testdirectory.questdiagnostics.com/test/test-detail/6631/?cc=MASTER.

https://www.accp.com/docs/sap/Lab_Values_Table_PedSAP.pdf.

https://www.unboundmedicine.com/harrietlane/view/Harriet_Lane_Handbook/309269/all/TABLE_27_1:_Reference_Values.

https://pdfs.semanticscholar.org/7106/09b4b2d315e448b4267a49420e1080da25eb.pdf.

file:///Q:/PediatricBloodGasesCriticalCarePanelTransportandECMOAgeRelatedReferenceandcv103117%20(2).pdf.
