# Supplementary material for: External evaluation of the Dynamic Criticality Index: A machine learning model to predict future need for ICU care in hospitalized pediatric patients
Source: PLoS One. 2024 Jan 29;19(1):e0288233. doi: 10.1371/journal.pone.0288233 (PMC10824440; doi:10.1371/journal.pone.0288233)
Supplement: S4 File — Percent accuracy of predictions for patients in the test sample who transferred from routine care to ICU care (n = 124) at fixed sensitivities of 0.85, 0.90, and 0.99 (a), and from ICU to routine care (n = 478) at fixed specificities of 0.85, 0.90, and 0.99 (b) for the Institutional Models of the Criticality Index-Dynamic. (PDF) [file pone.0288233.s004.pdf]

Supplemental information 4. Percent accuracy of predictions for patients in the test sample who transferred from routine care to ICU care (n = 124) at fixed sensitivities of 0.85, 0.90, and 0.99(a), and from ICU to routine care (n=478) at fixed specificities of 0.85, 0.90, and 0.99 (b) for the Institutional Models of the Criticality Index-Dynamic. The denominator varies by model because patients were required to be cared for in a location prior to transfer and new data in a time period.

Prediction Time Period Prior to Transfer from Routine to ICU Care at 85% sensitivity	Accuracy
>6 - 12 Hours	51.6% (64/124)
>12 - 18 Hours	50.6% (40/79)
>18 - 24 Hours	48.3% (28/58)
>24-30 Hours	50.0% (23/46)

Prediction Time Period Prior to Transfer from Routine to ICU Care at 90% sensitivity	Accuracy
>6 - 12 Hours	59.7% (74/124)
>12 - 18 Hours	75.9% (60/79)
>18 - 24 Hours	81.0% (47/58)
>24-30 Hours	73.9% (34/46)

Prediction Time Period Prior to Transfer from Routine to ICU Care at 99% sensitivity	Accuracy
>6 - 12 Hours	89.5% (111/124)
>12 - 18 Hours	91.1% (72/79)
>18 - 24 Hours	91.4% (53/58)
>24-30 Hours	91.3% (42/46)

Prediction Time Period Prior to Transfer from ICU to Routine care at 85% Specificity	Accuracy
>6 - 12 Hours	19.0% (91/478)
>12 - 18 Hours	31.4% (144/458)
>18 - 24 Hours	29.8% (123/413)
>24-30 Hours	39.1% (146/373)

Prediction Time Period Prior to Transfer from ICU to Routine care at 90% Specificity	Accuracy
>6 - 12 Hours	31.2% (149/478)
>12 - 18 Hours	44.5% (204/458)
>18 - 24 Hours	48.4% (200/413)
>24-30 Hours	55.5% (207/373)

Prediction Time Period Prior to Transfer from ICU to Routine care at 99% Specificity	Accuracy
>6 - 12 Hours	91.4% (437/478)
>12 - 18 Hours	93.7% (144/458)
>18 - 24 Hours	94.2% (123/413)
>24-30 Hours	92.8% (346/373)
