# Supplementary material for: External evaluation of the Dynamic Criticality Index: A machine learning model to predict future need for ICU care in hospitalized pediatric patients
Source: PLoS One. 2024 Jan 29;19(1):e0288233. doi: 10.1371/journal.pone.0288233 (PMC10824440; doi:10.1371/journal.pone.0288233)
Supplement: Attachment — Submitted filename: Author response4-11.docx [file pone.0288233.s005.docx]

Dear Editor-in-Chief,

I am re-submitting the original research article titled: **“External Evaluation of the Dynamic Criticality Index: A Machine Learning Model to Predict Future Need for ICU Care in Hospitalized Pediatric Patients”** by Patel et. al after a second revision and detailed response to reviewer 3. We appreciate your consideration for publication in *PLOS ONE* and the thoughtful editorial review that we believe has now greatly strengthened this manuscript. This will be the first ever publication to externally evaluate a pediatric machine learning algorithm developed from multi-institutional data that predicts future inpatient care needs in a single site; and compare these results to a single-site model developed using identical modelling methods and variables.

We believe our research is best suited for the *PLOS ONE* because the knowledge garnered from this research can positively influence and inform sound methods for evaluation of pediatric prediction algorithms that use novel machine learning methodology. This can have particularly important implications for models developed and intended for future clinical use as our results suggest that models developed from multi-institutional datasets should be assessed at individual institutions and may benefit from re-development with site-specific data prior to clinical deployment

We confirm that this manuscript has not been published elsewhere, is not under consideration by another journal, nor has the data been presented in another format such as abstract/poster/presentation. All authors have participated in the concept and design, analysis, and interpretation of data, in addition to drafting, reviewing, and/or revision of the manuscript. All authors have approved the final manuscript and agree with its submission to your journal.

A point by point response to reviewers is included below.

Please let me know of your decision at your earliest convenience.

With my best regards,

Anita K Patel, MD

Corresponding Author

Department of Pediatrics, Division of Pediatric Critical Care, Children’s National Health System and the George Washington University School of Medicine and Health Sciences, Washington DC

111 Michigan Ave NW

Washington, DC 20011

[apatel4@childrensnational.org](mailto:MPollack@childrensnational.org)

202-476-6817

**General Comments**:

**We are pleased that we seemed to have successfully responded to Reviewers 1 and 2 and hope that these comments are satisfactory to Reviewer 3.**

Please note that the overall goal of this analysis is to demonstrate that multi-institutional databases, often considered the ‘gold standard” for models, may loose performance in single sites. This performance issue can be overcome by applying the modeling lessons learned from multi-institutional models but applied to single sites. Reviewer #3 is focusing on more basic modeling issues which have been explored in manuscript references 8, 9, 10, and 18, not the issues related to bias secondary to the databases.

**Reviewer #3:**

1. **The goal of the paper is evaluating CI-D model, and assessing the single site performance of CI-D developed from a multi-institutional data base, and introduce neural networks to predict future need for ICU care****.** **Is the neural network intended to help evaluate CI-D model?**

We are sorry for this reviewer’s confusion. The goal of the manuscript is stated in the last sentence of the introduction (with minor edits, see below): “We performed this study to 1) assess the performance of the CI-D models developed from a multi-institutional database to predict future care locations when applied to a single site that did not participate in the multi-institutional dataset, (10) and 2) to apply the identical variables and modeling methods to a single-site dataset to develop models optimized for the single site, and compare this performance to the multi-institutional CI-D models’ performances.’

The purpose of the manuscript is not to "introduce neural networks to predict future need for ICU care.” Neural networks are the methodology used to derive all models used in the manuscript. To clarify the reviewer’s question, “Is the neural network intended to help evaluate CI-D model?” we did the following:

1. There is confusion between the use of the terms models, neural networks and algorithms. We eliminated the term “algorithms” and replaced it with “models” throughout the manuscript as it is unnecessarily confusing.
2. We clarified the issue of model and neural network as follows: Each prediction time-horizon (6 hrs – 12 hrs, etc.) has its own model, developed from separate neural networks. This is noted in Introduction>Para 2> “Four separate models were developed for prediction of care location in each of the four future time intervals.” To emphasize and clarify this issue that is confusing, we (a) revised Intro>Para 2>sentence 1 to: “Recently, we developed a set of four models developed from separate neural networks that predict future care needs assessed as care location using a new severity measure, the Dynamic Criticality Index (CI-D).(8)^,^(9)^,^(10)” (b) We added “models were” to Intro>Para 2> Sentence 3. (c) The last sentence of this paragraph has been clarified as “four models developed from separate neural networks.” (d). The last sentence of the Introduction now reads: “ We performed this study to 1) assess the performance of the CI-D models developed from a multi-institutional database to predict future care locations when applied to a single site that did not participate in the multi-institutional dataset,(10) and 2) to apply the identical variables and modeling methods to a single-site dataset to develop models optimized for the single site, and compare this performance to the multi-institutional CI-D models’ performances.”
2. **The contribution of the paper is confusing. It’s okay to evaluate CI-D model and tell readers where does CI-D model perform well and where does not, however, what can reader learn from the neural network? Is it a new proposed method to evaluate another algorithm? Please modify the introduction and declare the contributions.**

Please see response to #1 and General Comments. We hope the revisions proposed in #1 are helpful. Additionally, we have amended the final sentence of the introduction for clarity: “We performed this study to 1) assess the performance of the CI-D models developed from a multi-institutional database to predict future care locations when applied to a single site that did not participate in the multi-institutional dataset,(10) and 2) to apply the identical variables and modeling methods to a single-site dataset to develop models optimized for the single site, and compare this performance to the multi-institutional CI-D models’ performances.”

3. **The machine learning model and the application part has flaws and are not well explained:
   a) Why there are four separate model, and why these four intervals? (I believe there is some medical support behind, but please emphasize in the paper).**

There are four separate models developed using neural networks to predict care needs represented by care locations (ICU and non-ICU) in 4 different time periods in the future (0-6, 6-12, 12-18. 18-24 hours in the future). There are four models for each future prediction because that method had optimal prediction performance. We have now added the following statements for clarity:

a) To address the four 6-hour time period choice, paragraph one of machine learning methodology includes: “Six hours was selected because data acquisition for non-ICU care patients is relatively infrequent compared with ICU patients. A 6-hour time periods allowed for sufficient time to obtain a set of vital signs, and frequently a medication and/or lab result to inform future care location predictions.”
b) To address the relevance of the 4 future time periods and four separate models, the following has been added to paragraph 2 of the machine learning methodology section: “The four future time periods were chosen as clinically relevant time periods for both immediate and future resource allocation planning for potential increases and decreases in patient care needs. Separate models for each time period were used to optimize performance.”
c) To address the issue of the four separate models, we added the phrase “to optimize performance for each time period” to the first sentence of Machine Learning>Para 2 which now reads: “Independent neural networks calibrated to risk of ICU care were developed for four future time periods (>6-12 hours, >12-18 hours, >18-24 hours, and >24-30 hours) to optimize performance for each time period.”

**b) What are the neural networks predicting? Is the patient outcome a classification problem or regression (I assume classification because AUROC and AUPRC are the evaluation metrics, but please make it clear in the paper).**

We believe that readers with medical knowledge will not have this issue. However, we further clarified this issue. The second paragraph of the section ‘Machine learning methodology’ includes the following sentence clarifying that that the models are a classification problem rather than a regression: “A single neural network for binary classification for ICU outcome was developed for each future prediction time-period.”

We have further emphasized that the neural networks are classifying care location in the third paragraph of the ‘Machine learning methodology’ section, first sentence: “The single-site CI-D models’ classification performance was first assessed in the test sample using the same performance metrics detailed above.”

**c) The authors declared that the objective of teach training epoch was to maximize the Mathew correlation coefficient and minimize the cross entropy between predicted score and the patient’s outcome, however, it’s still not clear what is the loss/cost function? Is it cross entropy only, or a joint loss of cross entropy and (reversed) correlation coefficient?**

The methodology is detailed in referenced manuscripts (see references 8, 9, 10, 18) and only summarized in this one. To clarify the reviewer’s questions regarding model training: at every epoch, we attempted to minimize the cross entropy of the training set while monitoring the Mathew Correlation Coefficient (MCC) of the validation and the training sets. The numerical method we used to minimize cross entropy adds an additional step towards attaining a (possible local) minimum of the training cross entropy function. After this numerical step, we computed the MCC of both the training and validation sets. We strived to maximize the MCC of the validation set while monitoring the MCC of the training set.

The training loss/cost function is a mixture of both the MCC and the cross entropy. The minimization algorithm works on the cross entropy; however it is accepted based on the behavior of the MCC of the validation and implicitly on its difference with the training MCC. After we achieved convergence (to a minimum), we assessed for overfitting and underfitting using the described additional derivations of confusion matrices on both the training and validation sets. The minimization of the training cross entropy, the maximization of validation MCC and its difference to the training MCC is a dynamic process. We minimized overfitting by using the derivations of confusion matrices at different cut points a posteriori. We assessed over fitting or underfitting by evaluating the trajectory plots of derivations of confusion matrices. We started the process of training again by using the weights up to the moment there was no overfitting/underfitting. The plot below is the after-the-fact check for overfitting/underfitting of a model trained with 240 epochs.

We have updated the maximization/minimization objective in the main document as follows: Machine Learning Methodology>Para 2: Amended sentence using the following sentence in paragraph 2 of methods: machine learning methodology:

***“The objective of each training epoch was to minimize the training binary cross entropy while monitoring local maximization of the validation Mathew correlation coefficient (MCC) at a cut point of 0.5 while not allowing for drift from the training MCC at same cut point.”***

**d) What does the data look like. Is it time-series? Discrete numeric features? other type of features?**

Note that this information is detailed in (and referenced to) previous manuscripts. The data is time series with both discrete numeric features and continuous values. Details of the dataset and elements included in the model are in the appendices 1-3 of the manuscript. For reference, here is a table from appendix 1:

| **Lab Variables^1,3,4^** | | | | **Vital Signs^1,3,4^** | **Medications^2,3^** | **Other** |
| --- | --- | --- | --- | --- | --- | --- |
| Albumin | Bilirubin Indirect | Hemoglobin | Platelets | BP-systolic | 1113 individual medications | Age  Sex |
| ALT | Bilirubin Total | Hematocrit | Potassium | BP- diastolic | 143 medication categories (6) |  |
| Arterial Lactate | BUN | INR | Protime | Heart Rate |  |  |
| PO2 (arterial) | Calcium | Glucose | Sodium | Respiratory Rate |  |  |
| AST | Calcium Ionized | PTT | Total Protein | Temperature |  |  |
| Base Excess | Chloride | PCO2^5^ | Venous Lactate | Coma Score |  |  |
| Bicarbonate | Creatinine | pH^5^ | WBC |  |  |  |
| Bilirubin Direct | Fibrinogen |  |  |  |  |  |
| 1. Summarized for modeling with the following statistics for each variable: the count, sample mean, sample standard deviation (0 if the count was <2), maximum, and minimum. There were a total of 934 variables used for modeling: 300 derived from the 30 laboratory variables, 60 from the 6 vital signs, and 572 from the 143 medication categories, sex and age of patient at admission. 2. Summarized for modeling with the following statistics: the 6-hour sum per medication category of the number of medications given each hour; 2) the count of the previous time periods per medication category that the patient received one or more medications; 3) the proportion of the previous time periods per medication category that the patient received one or more medications. 3. Therapeutic intensity is reflected in the number of vital sign and laboratory measurements and medications. 4. If during the first six-hour time period there were missing values, these values were adjusted to the median of the first six-hour time periods adjusted to the following age groups: <1week, 1week-<4weeks, 4weeks-<3months, 3months-<1year, 1year-<2years, 2years-<3years, 3years-<8years, 8years-<12years, 12years-<22years. 5. Arterial, venous, capillary. 6. Classified by Multum^1^ | | | | | |  |

**e) What exactly is the structure of the neural network? The authors started with a neural network with a single hidden layer, and tried many things later, however, did not declare their decision of the network, nor did they show which attempt helped and which did not.**

Again, this information is in the previous manuscripts and in the additional information provided above. The final structure of the neural network is explained in the response to the next question.

While it is not possible to explore all possible structures within the space of fully connected neural networks, we are confident that our final models are optimal for fully connected neural networks and less than 9 hidden layers. Some models with more than five hidden layers had similar performances to our final models, this was at the cost of large regularization parameters. We chose models that were as simple as possible while maintaining performances. The training process we described led us to the final models.

We explored different model spaces such as RNN, LSTM, GRU, CNN, achieving lower classification performances. It is not withing the scope of this paper to show these details.

**f) What neural network model is applied? Fully-connected network? RNN/LSTM? CNN?**

Note that this is not a methodology paper but an application manuscript for a medical journal. The goal is to alert readers for performance issues associated with machine learning models. Since these are beginning to be used in both medical research and practice, we believe this is a relevant topic.

The second paragraph of the section “Machine learning Methodology, ” last sentence reads:

***“******The final neural networks were fully connected with five sequential hidden layers, but different number of nodes across hidden layers and models. Each layer had L_1_, L_2_ and dropout node regularizations with varying smoothing parameters. Each model has an output layer with one node and logistic activation.”***

Also, second paragraph of the “Machine Learning Methodology” section reads:

***“If overfitting was found, we add L_1_ and L_2_ regularization, and layer node dropouts with parameters tuned to maintain similar metrices on the validation and training sets”***

Third paragraph of the same section reads:

***“If overfitting was found, we add L2 regularization, and layer node dropouts with parameters tuned to maintain similar metrices on the validation and training sets”***

4. **We previous suggested the authors to add other models to compare with: a). Traditional machine learning such as random forest, logistic regression. b). LSTM if time-series data. This is because neural networks do not always perform better than traditional machine learning models. Also, if the data is time-series, LSTM is a better neural network than fully-connected networks.**

As noted above, this is not a methodology paper but an application manuscript for a medical journal. The goal is to alert readers for performance issues associated with machine learning models. Since these are beginning to be used in both medical research and practice, we believe this is a relevant topic.

For the reviewer’s reference, we did explore other modeling approaches prior to arriving at the current approach. As the reviewer suggested, we trained basic recurrent neural networks, gru, and lstm architectures, however these models had lower overall performance. To ensure that the model accounted for past clinical data in future predictions, we explicitly provide 24 hours of prior data for each patient as inputs in our neural networks. This methodology resulted in the best performing models among the different modeling methods we attempted.

5. **The way to design the network is ad-hoc. The authors tried a few random things and search which works the best, however, is not an ideal way of designing neural networks. The design of a network needs reasons and domain knowledge support. Trying many things cannot tell readers any useful information when they want to apply the paper to their own work, and there are always new techniques to try.**

As noted above, this is not a methodology paper but an application manuscript for a medical journal. The goal is to alert readers for performance issues associated with machine learning models. Since these are beginning to be used in both medical research and practice, we believe this is a relevant topic. The issue in the manuscript is not “how to design a neural network” but the performance of the network developed on what is often considered ”gold standard” data (multi-institutional data) in a new data set. The logic behind the evolution of this network is contained in the series of papers that preceded it. The focus on this paper is how the medical issues influenced the networks, more than the specifics of the networks. This is meant to be a manuscript for a medical audience.
