# Supplementary material for: External evaluation of the Dynamic Criticality Index: A machine learning model to predict future need for ICU care in hospitalized pediatric patients
Source: PLoS One. 2024 Jan 29;19(1):e0288233. doi: 10.1371/journal.pone.0288233 (PMC10824440; doi:10.1371/journal.pone.0288233)
Supplement: Attachment — Submitted filename: author response-5-3.docx [file pone.0288233.s006.docx]

Dear Editor-in-Chief,

I am re-submitting the original research article titled: **“External Evaluation of the Dynamic Criticality Index: A Machine Learning Model to Predict Future Need for ICU Care in Hospitalized Pediatric Patients”** by Patel et. al after a third revision and detailed response to reviewer 3. We appreciate your consideration for publication in *PLOS ONE* and the thoughtful editorial review that we believe has now greatly strengthened this manuscript. This will be the first ever publication to externally evaluate a pediatric machine learning algorithm developed from multi-institutional data that predicts future inpatient care needs in a single site; and compare these results to a single-site model developed using identical modelling methods and variables.

We believe our research is best suited for the *PLOS ONE* because the knowledge garnered from this research can positively influence and inform sound methods for evaluation of pediatric prediction algorithms that use novel machine learning methodology. This can have particularly important implications for models developed and intended for future clinical use as our results suggest that models developed from multi-institutional datasets should be assessed at individual institutions and may benefit from re-development with site-specific data prior to clinical deployment

We confirm that this manuscript has not been published elsewhere, is not under consideration by another journal, nor has the data been presented in another format such as abstract/poster/presentation. All authors have participated in the concept and design, analysis, and interpretation of data, in addition to drafting, reviewing, and/or revision of the manuscript. All authors have approved the final manuscript and agree with its submission to your journal.

A point by point response to reviewers is included below.

Please let me know of your decision at your earliest convenience.

With my best regards,

Anita K Patel, MD

Corresponding Author

Department of Pediatrics, Division of Pediatric Critical Care, Children’s National Health System and the George Washington University School of Medicine and Health Sciences, Washington DC

111 Michigan Ave NW

Washington, DC 20011

[apatel4@childrensnational.org](mailto:MPollack@childrensnational.org)

202-476-6817

**General Comments**:

**We are pleased that we seemed to have successfully responded to Reviewers 1 and 2 and hope that these responses to Reviewer 3’s final modelling questions appropriately address all remaining concerns.**

**1. Line 210 "at cut points of 0.15, 0.5, and 0.9 in the training and validation sets": it's not very clear what are these cutting points about.**

Thank you for this clarifying question. We have now emphasized the definition of the cut points in relation to the output of the neural networks in following locations: (lines in red are new edits to the manuscript, whereas lines in black were already present but pasted below for context)

Line 202: “A single neural network for binary classification for ICU outcome was developed for each future prediction time-period. The raw output of the neural networks was a continuous number between 0 and 1 which was converted to a binary classifier (ICU care or non-ICU care) by choosing a cut point. A neural network output below the set cut point was classified as non-ICU care, whereas an output above the set cut point was classified as ICU care.”

Line 208: “We started with a neural network with a single hidden layer, and logit output and consecutively increased the number of nodes while monitoring for overfitting by computing the MCC, sensitivity, specificity, precision, negative predictive value at cut points of 0.15, 0.5, and 0.9 for binary classification in the training and validation sets.”

**2. I don't think it's appropriate to say "if there was not overfitting". Overfitting will happen as the model is being trained, and it's important to catch the well-trained model before overfitting. E.g., using a validation and a test set - save the best performed model in validation and test it on test set.**

To address this concern, we have now amended this section as follows:

Line 208: “We started with a neural network with a single hidden layer, and logit output and consecutively increased the number of nodes while monitoring for overfitting by computing the MCC, sensitivity, specificity, precision, negative predictive value at cut points of 0.15, 0.5, and 0.9 for binary classification in the training and validation sets.”

**3. Line 219 "with minibatches of 10,000": is it 10,000 batches or with batch size 10,000. Also, the paper of citation 19 uses minibatch of size ten.**

We have now amended Line 220 as follows: “An initial learning rate of 0.0002 was used with minibatches each consisting of a size of 10,000 data points.”

The reference that the reviewer is referring to regarding minibatches was provided as a reference for the technique we implemented in our model development, not the absolute value of the minibatch size.
